# Surface electrostatics dictate RNA-binding protein CAPRIN1 condensate concentration and hydrodynamic properties

**DOI:** 10.1016/j.jbc.2022.102776

**Published:** 2022-12-07

**Authors:** Yuki Toyama, Atul Kaushik Rangadurai, Julie D. Forman-Kay, Lewis E. Kay

**Affiliations:** 1 Department of Molecular Genetics, University of Toronto, Toronto, Ontario, Canada; 2 Department of Biochemistry, University of Toronto, Toronto, Ontario, Canada; 3 Department of Chemistry, University of Toronto, Toronto, Ontario, Canada; 4 Hospital for Sick Children, Program in Molecular Medicine, Toronto, Ontario, Canada

**Keywords:** biomolecular condensates, NMR, intrinsically disordered protein, electrostatics, phase separation, FDG, 2-deoxy-2-fluoro-d-glucose, PRE, paramagnetic relaxation enhancement, PROXYL, 2,2,5,5-tetramethylpyrrolidine-*N*-oxyl nitroxide, TEMPO, 2,2,6,6-tetramethyl-1-piperidinyloxy, TROSY, transverse relaxation optimized spectroscopy

## Abstract

Biomolecular condensates concentrate proteins, nucleic acids, and small molecules and play an essential role in many biological processes. Their formation is tuned by a balance between energetically favorable and unfavorable contacts, with charge–charge interactions playing a central role in some systems. The positively charged intrinsically disordered carboxy-terminal region of the RNA-binding protein CAPRIN1 is one such example, phase separating upon addition of negatively charged ATP or high concentrations of sodium chloride (NaCl). Using solution NMR spectroscopy, we measured residue-specific near-surface electrostatic potentials (*ϕ*_ENS_) of CAPRIN1 along its NaCl-induced phase separation trajectory to compare with those obtained using ATP. In both cases, electrostatic shielding decreases *ϕ*_ENS_ values, yet surface potentials of CAPRIN1 in the two condensates can be different, depending on the amount of NaCl or ATP added. Our results establish that even small differences in *ϕ*_ENS_ can significantly affect the level of protein enrichment and the mechanical properties of the condensed phase, leading, potentially, to the regulation of biological processes.

The phase separation of biomolecules controls many aspects of cellular function (1, 2), and understanding the atomic details of the driving forces underlying this process has, therefore, been the subject of considerable efforts. The interactions that regulate the formation and disassembly of biological condensates have been extensively characterized *via* biochemical and computational studies (2, 3, 4); however, site-specific experimental information is lacking. Among the various interactions, the role of electrostatics is of particular interest as phase-separating proteins can be enriched in charged residues (5, 6), and altering protein surface electrostatics by interactions with charged molecules such as ATP or RNA (7, 8, 9) or *via* mutations or post-translational modifications (10, 11, 12) can dramatically affect a protein’s phase-separation propensity both *in vitro* and in cells. One prominent example where electrostatic interactions are critical to phase separation is provided by the 709 residue protein CAPRIN1, a molecule found in membraneless organelles, such as stress granules, P bodies, and mRNA transport granules that play important roles in regulating RNA processing (13, 14). We have previously demonstrated that the C-terminal 103-residue low-complexity region of CAPRIN1 (hereafter referred to as CAPRIN1) phase separates *in vitro*, and its relatively small size enables atomic-resolution NMR studies of the intramolecular and intermolecular interactions that drive its phase separation (15, 16, 17).

CAPRIN1 contains two Arg-rich sequences near the N and C termini (Arg-rich regions), with a middle region enriched in aromatic residues (aromatic-rich region). Since CAPRIN1 has 15 Arg residues, a pI of 11.5, and a charge of +13 at physiological pH, phase separation only occurs upon screening of unfavorable electrostatic interactions between positively charged CAPRIN1 chains; *in vitro* this can be achieved through the addition of charged molecules such as ATP or RNA or by the addition of high concentrations of sodium chloride (NaCl) (>600 mM for a 300 μM CAPRIN1 concentration, see later) (15, 16, 17). In order to understand how electrostatics regulate a particular biological process, a quantitative evaluation of the surface electrostatic potentials of the molecular players involved is required, which is typically achieved by solving the Poisson–Boltzmann equation using high-resolution structures of the proteins of interest (18, 19). However, the application of such a computational approach is fraught with difficulties for intrinsically disordered proteins such as CAPRIN1 that do not form well-defined structures. Recent advances in solution NMR spectroscopy provide an avenue for the experimental determination of per-residue near-surface electrostatic potentials (*ϕ*_ENS_) in such cases (20, 21, 22). The experiments are based on comparing contributions to transverse relaxation of NMR spin probes from added paramagnetic cosolutes sharing similar chemical structures but with different charges, enabling *de novo* determination of *ϕ*_ENS_ under a wide range of solvent conditions.

We have recently applied the method to investigate how *ϕ*_ENS_ values of CAPRIN1 “evolve” along its phase separation trajectory as increasing concentrations of ATP are added to promote phase separation (13). In a low-salt buffer without ATP, in which CAPRIN1 does not phase separate (mixed state), *ϕ*_ENS_ values are large and positive, +20 to 40 mV, with maxima at the N- and C-terminal Arg-rich regions of the molecule. Upon addition of small amounts of ATP (≤0.8 mM), *ϕ*_ENS_ values begin to decrease, reflecting the binding of ATP to the N- and C-terminal Arg-rich regions that lead to screening of positive charges as a first step in the phase separation process (13). In the condensed phase, that under our conditions (3 mM bulk ATP used to prepare the condensed phase) leads to high concentrations of protein (33 mM) and nucleotide (162 mM), almost complete charge neutralization is achieved (*ϕ*_ENS_ ≈ 0 mV), with approximately five ATP molecules interacting with one CAPRIN1 chain. At higher nucleotide concentrations (>60 mM bulk ATP), the binding of ATP to the aromatic-rich moieties of CAPRIN1 leads to small negative potentials in these regions and is accompanied by dissolution of condensates and re-entrance into a mixed state. A strong correlation between reduction in electrostatic potential and enhanced intermolecular interactions, mainly involving the aromatic-rich region of CAPRIN1, was also established from intermolecular paramagnetic relaxation enhancement (PRE) experiments (13).

Previously, we have shown that the addition of NaCl also promotes phase separation of CAPRIN1, but at a higher concentration (>500–600 mM for a 400 μM CAPRIN1 solution) (16) compared with ATP (∼1–50 mM) (17). It is reasonable to expect that the addition of salt screens unfavorable electrostatic interactions between proximal CAPRIN1 chains in a manner analogous to ATP. However, we questioned whether the *ϕ*_ENS_ profile of CAPRIN1 in the presence of NaCl would mirror that of ATP since the modes of interaction of the two compounds with CAPRIN1 are quite different. For example, ATP can interact with the aromatic-rich regions and potentially compete with the self-association process (17, 23) since intermolecular interactions are stabilized by aromatic–aromatic contacts between moieties on adjacent chains (4, 17) that may well be disturbed by the binding of ATP. Furthermore, high concentrations of NaCl are proposed to enhance favorable nonpolar-type intermolecular interactions (24). Thus, the degree of neutralization required for CAPRIN1 phase separation with NaCl and the nature of the intermolecular interactions that are formed might be different compared with ATP. If this is the case, then it is likely that the material properties of the condensate would also change. To address these possibilities, we have mapped out CAPRIN1 *ϕ*_ENS_ profiles as a function of NaCl concentration and characterized the intermolecular interactions and dynamics of CAPRIN1 in the salt-induced condensed phase. Notably, differences were found between the electrostatic potentials of salt and ATP-induced condensate samples that, in turn, give rise to differences in their material properties.

## Results and discussion

**Figure 1:**
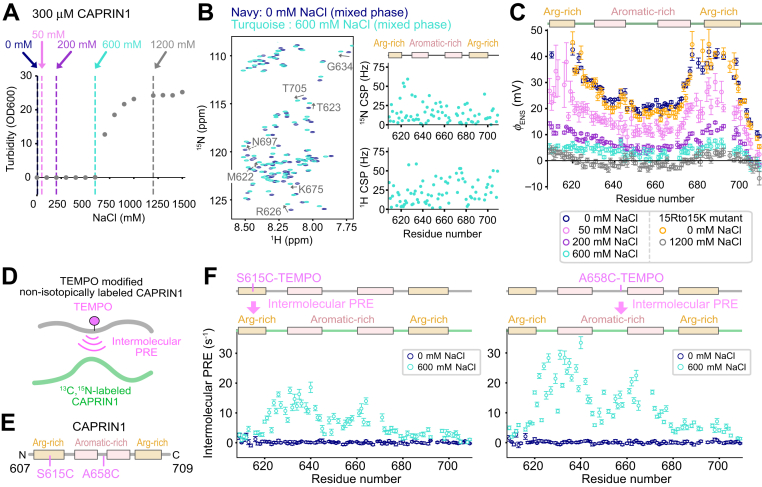
**Effect of salt on CAPRIN1 *ϕ***_**ENS**_**potentials and intermolecular interactions.***A*, turbidity assays measuring phase separation propensities of 300 μM samples of CAPRIN1 as a function of [NaCl]. The *vertical dotted lines* indicate the data points with 0 mM (*navy*), 50 mM (*pink*), 200 mM (*purple*), 600 mM (*turquoise*), and 1200 mM (*gray*) NaCl used in the NMR experiments. Error bars (smaller than data points) were estimated from triplicate measurements. *B*, *left*, overlay of ^15^N–^1^H TROSY HSQC spectra measured in the presence of 0 mM (*navy*) and 600 mM (*turquoise*) NaCl. *Right*, plots of the ^15^N (*top*) or ^1^H (*bottom*) chemical shift differences (absolute values) upon addition of 600 mM NaCl. *C*, plots of *ϕ*_ENS_ values measured with 0 mM (*navy*, CAPRIN1; *orange*, 15Rto15K CAPRIN1), 50 mM (*pink*), 200 mM (*purple*), 600 mM (*turquoise*), or 1200 mM (*gray*, 15Rto15K CAPRIN1) NaCl, in 300 μM CAPRIN1 mixed phase samples. *ϕ*_ENS_ potentials were determined by using 3-aminomethyl-PROXYL and 3-carboxy-PROXYL. Several *ϕ*_ENS_ values are missing for the N-terminal residues of the 0 mM salt samples, as the positively charged PROXYL derivative gives very small PRE values in this region. In the 1200 mM NaCl dataset, the 15Rto15K mutant of CAPRIN1 was used to suppress condensate formation. In the 50 mM NaCl dataset, the fraction of ^13^C,^15^N-labeled CAPRIN1 was reduced to 25% of the total protein, with the remaining 75% unlabeled, accounting for the larger errors. *D*, schematic showing TEMPO-modified and nonisotopically labeled CAPRIN1 chains and isotopically labeled CAPRIN1 used to measure the intermolecular PRE effect and hence the proximity of the spin label to amide protons of an adjacent NMR active molecule. *E*, positions of the Cys mutations used for the intermolecular PRE experiments (one at a time), and hence the location of the spin labels, are indicated. *F*, intermolecular PRE profiles for S615C-TEMPO (*left*) and A658C-TEMPO (*right*) mixed phase samples, comprising 150 μM ^12^C,^14^N-TEMPO CAPRIN1 and 150 μM ^13^C,^15^N CAPRIN1, are shown. The data recorded without NaCl (*navy*) and with 600 mM NaCl (*turquoise*) are overlaid in each plot. All the NMR data were recorded at pH 5.5, 3% D_2_O, 25 °C, and 1 GHz. Errors in *C* and *F* were obtained by propagating uncertainties in *R*_2_ rates. PRE, paramagnetic relaxation enhancement; PROXYL, 2,2,5,5-tetramethylpyrrolidine-*N*-oxyl nitroxide; TEMPO, 2,2,6,6-tetramethyl-1-piperidinyloxy; TROSY, transverse relaxation optimized spectroscopy.

In order to evaluate the concentration of NaCl required for phase separation of a 300 μM CAPRIN1 sample (pH 5.5, room temperature), we performed turbidity assays as a function of salt (Fig. 1*A*). The increase in light scattering that accompanies condensate formation shows that NaCl concentrations in excess of approximately 600 mM are required, consistent with our previous results that high concentrations of salt are necessary to promote phase separation (16). We first measured per-residue *ϕ*_ENS_ values at four different NaCl conditions, 0, 50, 200, and 600 mM, where CAPRIN1 is in the mixed state (*i.e*., not phase separated) to establish how *ϕ*_ENS_ evolves during the salt-induced phase separation trajectory. Following the procedure established by Yu *et al.* (21), and as described in our previous study (13), solvent PREs of backbone amide protons (^1^H^N^) were quantified from transverse relaxation rates measured using the differently charged 2,2,5,5-tetramethylpyrrolidine-*N*-oxyl nitroxide (PROXYL) derivatives: 3-aminomethyl-PROXYL (+1) and 3-carboxy-PROXYL (−1). Per-residue *ϕ*_ENS_ values were calculated from the relation (21).

(1) $$\phi_{ENS}=-\frac{k_{B}T}{2e}\ln\left(\frac{\Gamma_{2,+}}{\Gamma_{2,-}}\right)$$

where *k*_*B*_ is Boltzmann's constant, *T* is the absolute temperature, $\Gamma_{2,i}$ (i∈{+,−} where “+” and “−” denote 3-aminomethyl-PROXYL and 3-carboxy-PROXYL compounds, respectively) are the measured ^1^H *R*_2_ PRE contributions, and *e* is the magnitude of the charge of an electron. Before embarking on these measurements, we compared NMR spectra of mixed state CAPRIN1 (300 μM) in the presence of 600 mM NaCl and in the absence of salt (Fig. 1*B*). The overall chemical shift changes were small (<0.06 and <0.6 ppm in the ^1^H and ^15^N dimensions, respectively), with no region clearly affected more than another, suggesting that the chemical shift is not a sensitive reporter of changes in surface electrostatics induced by NaCl. Figure 1*C* shows plots of per-residue *ϕ*_ENS_ profiles measured at a number of different NaCl conditions at pH 5.5 and 25 °C. As the NaCl concentration increases, *ϕ*_ENS_ values progressively decrease, clearly reflecting the screening of charge by the mobile salt ions (25). At 600 mM NaCl, where CAPRIN1 starts to phase separate, *ϕ*_ENS_ values are very small (+3.7 mV on average), suggesting that charge neutralization is required for CAPRIN1 phase separation, as observed previously with ATP (13). We were also interested in establishing whether even higher concentrations of salt would lead to complete neutralization of CAPRIN1 molecules in the mixed phase. At a concentration of 300 μM CAPRIN1, used in our NMR studies of mixed phase solutions, phase separation occurs at NaCl concentrations above 600 mM, leading to a significant reduction in the protein concentration that is observed in NMR spectra, as the condensed phase that sinks to the bottom of the NMR tube removes protein from solution. This hampers mixed phase *ϕ*_ENS_ measurements at high salt concentrations. We have, therefore, used a 15Rto15K mutant of CAPRIN1, where all the Arg residues are replaced with Lys to significantly attenuate the phase separation propensity while conserving the charge state of the protein, as previously reported (16). In the absence of salt, *ϕ*_ENS_ values of CAPRIN1 and 15Rto15K CAPRIN1 in mixed phase samples are similar (Fig. 1, compare *dark blue* and *orange*), whereas in the presence of 1200 mM NaCl, the 15Rto15K mutant was close to neutral (*ϕ*_ENS_ = −0.13 mV on average) (Fig. 1*C*, *gray*).

It is expected that intermolecular contacts would increase as the CAPRIN1 surface charge decreases, since the repulsive electrostatic interactions between neighboring chains are reduced. To verify this, we carried out PRE experiments on mixed state samples whereby spin labels were covalently attached to NMR silent (*i.e.*, nonisotopically-labeled) molecules and the effect on amide proton relaxation rates in ^13^C,^15^N-labeled molecules quantified (Fig. 1*D*), using a 1:1 molar ratio of spin-labeled and unmodified proteins. Spin labels were introduced by making single Cys mutations (S615C and A658C), with the subsequent covalent attachment of the 2,2,6,6-tetramethyl-1-piperidinyloxy (TEMPO) radical at these Cys sites *via* a thiol–maleimide linkage. S615 and A658 are in the Arg-rich region near the N terminus and the middle aromatic-rich region of CAPRIN1, respectively, both of which are involved in intermolecular CAPRIN1 contacts that are important in stabilizing the condensate (Fig. 1*E*) (13, 17). Intermolecular PREs were quantified from differences in ^1^H^N^ *R*_2_ relaxation rates measured for the paramagnetic and diamagnetic samples comprising 150 μM ^13^C,^15^N CAPRIN1, and 150 μM TEMPO-labeled/nonisotopically-labeled CAPRIN1 (TEMPO was reduced by adding ascorbate to form the diamagnetic sample). Figure 1*F* shows the intermolecular PRE patterns from S615C-TEMPO and A658C-TEMPO samples measured in the absence or the presence of 600 mM NaCl. In the absence of NaCl, intermolecular PREs were not observed, reflecting the electrostatic repulsion between CAPRIN1 chains that prevents close interactions. In contrast, significantly increased PRE values were measured with 600 mM NaCl, indicating enhanced intermolecular interactions, that is, the spin label on a nonisotopically-labeled chain and the amide protons on an unmodified/^13^C,^15^N-labeled chain become closer in the presence of high salt. The PRE profiles from the two labeled sites are similar, with two maxima observed in the aromatic-rich portions of the molecule, consistent with results from our previous studies based on intermolecular NOEs measured in the condensed state (17). These results provide evidence, as before (13), that the aromatic regions are hot spots for interchain contacts that contribute to the stabilization of the condensate and that the interactions are degenerate, involving multiple regions of adjacent chains. We note that the PRE profiles are highly correlated to those measured in the presence of ATP (13), that is, regions with large PREs were very similar, and only the overall magnitude of the enhancement was different between salt- and ATP-generated condensates. These results strongly suggest that the intermolecular contacts responsible for condensate formation are preserved between salt- and ATP-induced phase separation.

**Figure 2:**
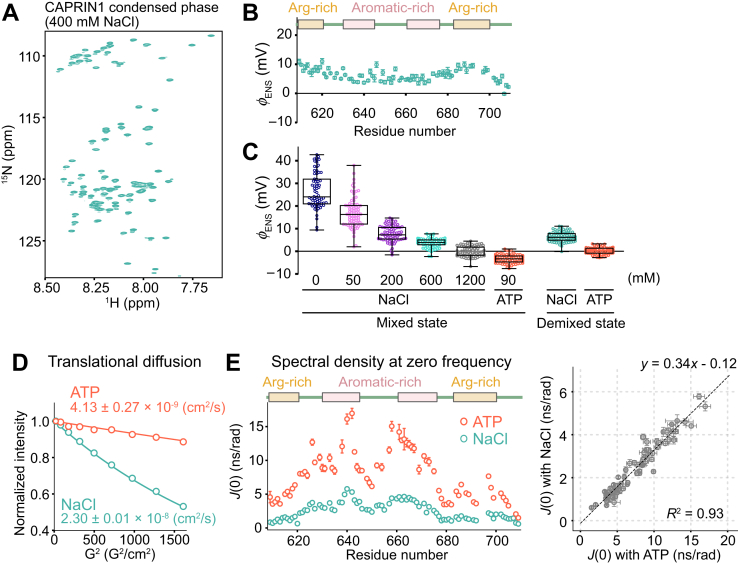
**Characterization of per-residue near surface electrostatic potentials and hydrodynamic properties of CAPRIN1 in the condensed phase.***A*, ^15^N–^1^H TROSY HSQC spectrum of a condensed phase CAPRIN1 sample generated by addition of 400 mM bulk sodium chloride (NaCl) (19.1 ± 1.5 mM protein). The measurement was performed at pH 5.5, 40 °C, and 1 GHz. *B*, per-residue CAPRIN1 *ϕ*_ENS_ values in the (400 mM) salt-induced condensed phase determined by using 3-aminomethyl-PROXYL and 3-carboxy-PROXYL spin labels. Errors were obtained by propagating uncertainties in *R*_2_ rates. *C*, box plots showing the distribution of *ϕ*_ENS_ values of mixed state CAPRIN1 samples (300 μM in protein), with various concentrations of NaCl or 90 mM ATP (*left*) and those in the condensed phase generated by addition of bulk concentrations of 400 mM NaCl or 3 mM ATP (*right*). The *line* within the *box* represents the median, and the outer edges of the box are the 25% and 75% percentiles of the values. The *whiskers* extend to the minimum and maximum values. All the measured datapoints (*i.e.*, all residues) are shown as *colored dots*. The 1200 mM NaCl dataset was measured using the 15Rto15K mutant of CAPRIN1 that does not phase separate under these conditions. *D*, measurements of translational diffusion coefficients of CAPRIN1 in condensed phases generated by the addition of bulk concentrations of 400 mM NaCl (*teal*) or 3 mM ATP (*orange*). Diffusion measurements were performed at pH 5.5, 40 °C, and 800 MHz using a pulse scheme described previously (29). Errors were computed from the standard error of the fit. *E*, *left*, plots of per-residue spectral densities evaluated at zero frequency, *J*(0), in the salt-induced (*teal*) or ATP-induced (*orange*) condensed phases of CAPRIN1. *Right*, linear correlation plot of the *J*(0) values. The measurements were performed at pH 5.5, 10% D_2_O, 40 °C, and 1 GHz. Errors in *J*(0) were computed by propagating the standard error of the fit of each relaxation rate and heteronuclear NOE value. PROXYL, 2,2,5,5-tetramethylpyrrolidine-*N*-oxyl nitroxide; TROSY, transverse relaxation optimized spectroscopy.

It is important to emphasize that the *ϕ*_ENS_ measurements described previously (Fig. 1*C*) were carried out on mixed state samples. The small amount of condensate that is formed at 600 mM NaCl simply decreases the fraction of observable signal derived from the dilute phase. Therefore, the electrostatic potential measured at 600 mM salt, corresponding to the onset of phase separation, is not the potential within condensed phase droplets. In order to obtain residue-specific *ϕ*_ENS_ values of CAPRIN1 molecules in the condensed phase, we have prepared a demixed state sample at a bulk NaCl concentration of 400 mM, used previously for the measurement of intermolecular and intramolecular interactions in the condensed phase (17). Note that for the high concentrations of protein used to generate phase-separated samples, 400 mM salt is sufficient, in contrast to much lower CAPRIN1 concentrations where higher salt is required (Fig. 1*A*). Two distinct liquid phases were present in the NMR sample, including a CAPRIN1-depleted phase on top (dilute phase; 3.2 ± 0.6 mM in protein) and a CAPRIN1-concentrated phase on the bottom (condensed phase; 19.1 ± 1.5 mM), either without or with a PROXYL spin-label derivative. As described previously (13, 17), it is straightforward to ensure that only the condensed phase is detected by positioning the bottom region of the sample in the NMR receiver coil, with the dilute phase well outside the coil boundaries. Figure 2*A* shows an ^15^N–^1^H transverse relaxation optimized spectroscopy (TROSY) (26) heteronuclear single quantum coherence spectrum of CAPRIN1 in the condensed phase recorded at 40 °C using a sample containing 2% ^2^H,^15^N-labeled protein in an unlabeled background in the absence of paramagnetic cosolutes. Solvent PREs and subsequently *ϕ*_ENS_ values were determined from such TROSY-based spectra recorded with aminomethyl-PROXYL and carboxy-PROXYL cosolutes (one at a time) or no cosolute (Fig. 2*B*). The distribution of *ϕ*_ENS_ values so obtained was compared with those measured using the mixed state samples containing various concentrations of NaCl or 90 mM ATP, as well as values from the ATP-induced condensed phase (3 mM bulk ATP), reported previously (13) (Fig. 2*C*). The *ϕ*_ENS_ values from the condensed phase (400 mM bulk NaCl) were significantly lower than those measured in the mixed state in the absence of salt, reflecting the significant electrostatic shielding effect by the mobile salt ions in the condensate. Notably, however, the *ϕ*_ENS_ values were mainly distributed between +5 and +10 mV, indicating that CAPRIN1 is slightly positively charged in the condensed phase. Very similar, slightly positive per-residue electrostatic potentials were also observed when the experiments were repeated at 25 °C. This is in sharp contrast to the ATP-induced condensed phase where CAPRIN1 was almost completely neutral (*ϕ*_ENS_ ∼ 0 mV), becoming slightly negatively charged (*ϕ*_ENS_ ∼ −3 to −4 mV) with the dissolution of the condensed phase upon further addition of ATP (90 mM bulk) (13).

We were interested in establishing whether the difference in the surface electrostatic potentials between the salt- and ATP-induced condensed phases would lead to changes in the dynamics of CAPRIN1 chains and in the material properties of the condensates. To this end, we first performed pulsed-field gradient NMR experiments to measure the translational diffusion constants of CAPRIN1 in the salt-induced and ATP-induced condensed phases at 40 °C (Fig. 2*D*). The diffusion constants of CAPRIN1 were 2.30 ± 0.01 × 10^−8^ cm^2^/s and 4.13 ± 0.27 × 10^−9^ cm^2^/s in the salt- and ATP-induced condensed phases, respectively, corresponding to translational diffusion rates that are approximately sixfold smaller in the ATP sample. We also carried out a series of backbone ^15^N dynamics measurements to evaluate motional properties in each condensate, separating the potential effects of chemical exchange from intrinsic relaxation properties (27). Figure 2*E* left plots the spectral density function evaluated at zero frequency, *J*(0) = 0.4 × *S*^2^τ_C_, where *S*^2^ quantifies the amplitude and τ_C_ the effective correlation time of the motion of each backbone amide bond vector on the picosecond-to-nanosecond timescale (27, 28). The *J*(0) values are about threefold larger in the ATP-induced condensed phase, suggesting that the effective rotational and/or segmental motion of each NH bond vector of CAPRIN1 is significantly slowed relative to the situation in salt. Notably, the patterns of *J*(0) values were highly correlated between the two condensed phase samples (*R*^2^ = 0.93, Fig. 2*E*, *right*). We have previously shown that the site-specific increase in *J*(0) values around the two aromatic-rich regions results from intermolecular contacts that are responsible for the stabilization of the condensate (13, 17). Thus, the strong correlation between *J*(0) values indicates that a similar set of residues is involved in the formation of CAPRIN1 interchain contacts in both condensed phase environments. It is of interest to note that the translational diffusion rate is attenuated more significantly than the *J*(0) values between salt- and ATP-driven condensed phases, indicating that molecular crowding affects translation more significantly. It is noteworthy that the concentrations of protein in either condensed phases (see later) are above the concentration at which the transition from a dilute to a semidilute solution occurs, corresponding to the regime where protein molecules directly contact each other (∼200 mg/ml for CAPRIN1). Thus, CAPRIN1 molecules constantly collide with each other as they move, significantly slowing translation, whereas segmental motions of the protein backbone or rotation are less affected.

What is the origin of the different CAPRIN1 properties in the two condensed phases? The concentration of CAPRIN1 in the condensed phase generated by addition of ATP at a bulk concentration of 3 mM is 32.9 ± 1.7 mM (13), approximately twofold higher than that measured in the 400 mM salt-induced condensed phase (19.1 ± 1.5 mM). The higher concentration of CAPRIN1 in the ATP-containing droplets increases their viscosity, leading to the slower rates of protein translational and rotational motion. We hypothesized that the amount of CAPRIN1 in the condensed phase is, in turn, inversely correlated to the degree of “residual” electrostatic repulsion between protein molecules as quantified by *ϕ*_ENS_ values. In this manner, the slight positive potential in the salt-induced condensate (∼+5–10 mV) would limit the incorporation of CAPRIN1 molecules relative to ATP-induced condensed phases where CAPRIN1 is almost completely neutral (∼0 mV) and intermolecular interactions, thus, more favorably formed. To test this idea, we measured the CAPRIN1 concentration in a condensate prepared with a higher concentration of NaCl (1200 mM), where *ϕ*_ENS_ values are expected to be close to 0 mV, as measured for the 15Rto15K mutant in the mixed state at 1200 mM salt (Fig. 1*C*). Notably, the concentration of CAPRIN1 was significantly increased, from 19.1 ± 1.5 mM (400 mM bulk NaCl) to 38.8 ± 1.6 mM (1200 mM bulk NaCl), comparable to the value measured in the ATP-induced condensate (32.9 ± 1.7 mM at 3 mM bulk ATP). This result is also in line with phase diagrams that we have generated, in which the condensed phase CAPRIN1 concentration significantly increases with the concentration of salt (17). Taken together, our data suggest that the CAPRIN1 electrostatic potential within the condensate closely regulates protein concentration, and, hence, the material properties of the condensate, and that the extent of modulation of *ϕ*_ENS_ depends on the concentration of each of the neutralizing anions, with ATP more effective than chloride because of its more negative charge.

## Concluding remarks

**Figure 3:**
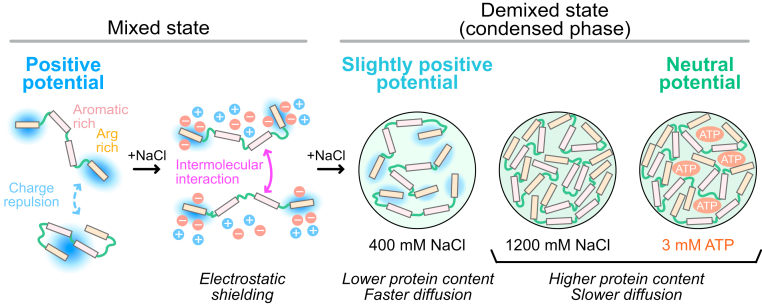
**The electrostatic potential of CAPRIN1 as a function of added sodium chloride (NaCl) and comparison of NaCl- and ATP-induced phases.** Schematic of the NaCl-induced phase separation trajectory of CAPRIN1, as described in the text. Addition of NaCl screens positive charges and promotes intermolecular interactions, ultimately leading to phase separation. Under the conditions of our experiments, the CAPRIN1 molecules are positively charged, *ϕ*_ENS_ ≈ +5 to 10 mV, in the condensed phase prepared using a bulk concentration of 400 mM sodium chloride, with additional salt (1200 mM bulk concentration) leading to further screening of charge and higher protein concentrations. Notably, protein concentrations and condensate viscosity are higher as the CAPRIN1 surface charge is neutralized, using either NaCl or ATP.

Figure 3 summarizes the results of this study schematically. CAPRIN1 in low-salt buffer has a large and positive surface electrostatic potential across the molecule (>+20 mV), with maxima at the N- and C-terminal Arg-rich regions of the protein (≈+40 mV), thus preventing self-association through charge–charge repulsion. Upon addition of increasing amounts of NaCl, the positive surface potential of CAPRIN1 becomes efficiently screened, leading to enhanced intermolecular interactions and, ultimately, to phase separation. The per-residue *ϕ*_ENS_ values in the salt-induced condensed phase prepared using 400 mM bulk NaCl establish that CAPRIN1 is slightly positive, in contrast to ATP-induced droplets prepared with 3 mM bulk ATP, where nearly complete neutralization of CAPRIN1 is achieved (13). Our intermolecular PRE results in the mixed state, and the spectral density analyses in the condensed phase collectively indicate that the intermolecular interactions responsible for stabilizing the condensate are highly conserved between the two different condensed phases, with the same set of residues participating in the formation of intermolecular contacts. Significant differences in protein concentrations in the salt and ATP condensates reflect differences in residual charges of CAPRIN1 molecules that, in turn, affect the material properties of the condensates. Our finding that even small changes in electrostatic potentials of a few millivolts can lead to changes in the properties of the condensed phase is significant. CAPRIN1 interacts with RNA and other negative binding partners, such as fragile X mental retardation protein that can be phosphorylated, and its net charge can be further perturbed *via* post-translational modifications (15). Such, potentially nuanced, changes to surface electrostatics could play an important role in dictating critical concentrations of components both inside and outside the condensate as well as the hydrodynamic properties of molecules in the condensed phase, as we have shown here, potentially regulating, for example, rates of enzymatic reactions.

## Experimental procedures

### Sample preparation

The C-terminal low-complexity region of CAPRIN1, extending from residues 607 to 709 (UniProt ID: Q14444), and its 15Rto15K variant, where all the 15 Arg in the sequence were replaced with Lys, were expressed and purified as described previously (13, 15, 16). Residues N623–G624 and N630–G631 slowly form isoaspartate–Gly peptide linkages over time, which can alter the charge distribution of CAPRIN1 and change intensities of correlations in the NMR spectra (16). To abolish isoaspartate formation, we introduced N623T and N630T double mutations and used the double Thr mutant in all the experiments described in this study (referred to as CAPRIN1 in what follows). N623T and N630T double mutations and Cys mutations (S615C and A658C) were introduced by using a QuikChange Site-Directed Mutagenesis Kit (Agilent). We have previously shown that the introduction of (i) the N623T and N630T double mutations or (ii) TEMPO to either S615C or A658C molecules does not perturb the phase separation propensities of CAPRIN1 (13).

Mixed state NMR samples consisted of 300 μM U–^13^C,^15^N CAPRIN1, 25 mM Mes–NaOH (pH 5.5), and 3% D_2_O, with or without 50 to 1200 mM added NaCl. The 15Rto15K mutant of CAPRIN1 was used to suppress phase separation, while simultaneously allowing measurement of electrostatic potentials at high salt (1200 mM NaCl). All demixed state samples were prepared as described previously (13, 15). Phase separation was induced by mixing ∼6 to 8 mM CAPRIN1 (2% ^2^H, ^15^N-labeled protein + 98% unlabeled protein) with 400 mM NaCl (bulk) in 25 mM Mes–NaOH (pH 5.5) and 10% D_2_O at room temperature. Care was taken to pH the CAPRIN1 solution after buffer exchange and concentration before adding salt to induce phase separation. The sample volumes were estimated after vortexing, and each PROXYL derivative and a 2-deoxy-2-fluoro-d-glucose (FDG) standard (FLUKA) added to concentrations of approximately 5 and 1 mM, respectively (FDG was added to the condensed phase samples to quantify the concentrations of added cosolute, as discussed later and in more detail previously (13)). After centrifugation, the bottom phase, corresponding to the condensed protein, was transferred to a 3 mm NMR tube.

Intermolecular PRE experiments were performed on CAPRIN1 samples prepared as described previously (13). Briefly, purified Cys-mutant CAPRIN1 was reduced by incubating with 5 mM DTT, after which the excess DTT was removed by buffer-exchanging the solution using a PD-10 desalting column (Cytiva). Proteins were then reacted with 10 equivalents of *N*-(1-oxyl-2,2,6,6-tetramethyl-4-piperidinyl)maleimide (4-maleimido-TEMPO) (Toronto Research Chemicals) overnight at 4 °C in buffer containing 25 mM sodium phosphate and 2 M guanidinium chloride. The reaction was quenched by adding 5 mM DTT, with subsequent removal of unreacted 4-maleimide-TEMPO by gel filtration using a HiPrep 26/10 Desalting column (Cytiva). The reduced state of the spin label was prepared by incubating TEMPO-labeled CAPRIN1 with 20 equivalents of l-ascorbic acid overnight at room temperature. The reduced sample was further purified by an additional gel-filtration step using a HiPrep 26/10 Desalting column to remove the excess l-ascorbic acid. Cys modifications were confirmed using electrospray ionization mass spectrometry.

### Turbidity assays

Purified CAPRIN1 samples were diluted to a protein concentration of 300 μM with 25 mM Mes–NaOH, pH 5.5 containing varying concentrations of NaCl (ranging from 0 to 1500 mM). About 5 μl samples were loaded into a μCuvette G1.0 (Eppendorf) after vigorous mixing, and absorbance measurements at 600 nm were recorded three times using a BioPhotometer D30 (Eppendorf).

### NMR measurements

All the ^1^H-detected NMR measurements were performed at 23.5 T (1 GHz ^1^H frequency) on a Bruker Avance Neo spectrometer or using a 18.8 T (800 MHz ^1^H frequency) Bruker Avance III HD spectrometer, equipped with cryogenically cooled *x*, *y*, and *z* pulsed-field gradient triple-resonance probes. The ^19^F-detected NMR measurements were performed at 11.7 T (500 MHz ^1^H frequency) on a Bruker Avance III HD spectrometer equipped with a liquid nitrogen-cooled *z* pulsed-field gradient triple-resonance probe. All measurement and analysis details are as described previously (13).

### Calculations of near-surface electrostatic potentials

Near-surface electrostatic potentials were calculated from *R*_2_ PRE rates obtained with a pair of PROXYL derivatives (Γ_2,*+*_ and Γ_2,−_) using Equation (1) as described previously (21), where *k*_B_ = 8.62 × 10^−5^ eV/K; *T* = 298.15 K or 313.15 K for the mixed or demixed state samples, respectively. In the calculations, only residues with PRE values larger than 0.5 s^−1^ were used. Concentrations of stock solutions of paramagnetic cosolutes used in preparing mixed state samples were estimated using the method of Yu *et al.* (21). We have taken a different approach for measuring concentrations of added cosolutes in the case of the demixed sample, described in detail previously (13). Briefly, possible differences in concentrations of cosolutes were accounted for by correcting ratios of PRE rates ($\Gamma_{2,+}^{corr.}/\Gamma_{2,-}^{corr.}$) using Equation (2)

(2) $$\frac{\Gamma_{2,+}^{corr.}}{\Gamma_{2,-}^{corr.}}=\frac{\Gamma_{2,+}^{\exp.}}{\Gamma_{2,-}^{\exp.}}\cdot \frac{\Gamma_{1,-}}{\Gamma_{1,+}}\cdot \frac{k_{+}}{k_{-}}$$

where $\Gamma_{2,+}^{\exp.}$ and $\Gamma_{2,-}^{\exp.}$ are experimentally measured ^1^H^N^ *R*_2_ PRE values and $\Gamma_{1,+}$ and $\Gamma_{1,-}$ are the water or FDG *R*_1_ PRE rates (*i.e.*, $\Gamma_{1,i}=R_{1}^{para.,i}-R_{1}^{dia.}$), which are proportional to the concentration of the spin label, measured in the condensed phase sample. *k*_*i*_ is the slope of $\Gamma_{1,i}$ as a function of the concentration of paramagnetic cosolute *i*, obtained from calibration experiments, which takes into account the fact that relaxation rates of either water or ^19^F in FDG may be affected by one cosolute differently than a second (at equivalent concentrations) (13). In this way, the ratio of *R*_2_ PREs between aminomethyl- and carboxy-PROXYL datasets ($\Gamma_{2,+}^{\exp.}/\Gamma_{2,-}^{\exp.}$) in the salt-induced condensed phase was corrected by multiplying by 1.64 $\left(=\frac{\Gamma_{1,-}}{\Gamma_{1,+}}\cdot \frac{k_{+}}{k_{-}}\right)$. The correction factor was significantly larger than the value used for the ATP-induced condensed phase in our previous study (13), which may reflect different amounts of the added paramagnetic cosolutes between the samples and/or the favorable (or unfavorable) partitioning of carboxy-PROXYL (or aminomethyl-PROXYL) into the salt-induced condensed phase.

### Translational diffusion measurements

The translational diffusion constants of CAPRIN1 in the condensed phase were measured using the stimulated-echo based pulse scheme described in the study by Choy *et al.* (29) with a slight modification to incorporate a gradient coherence selection scheme to eliminate artifactual aromatic signals from ATP samples that would otherwise interfere with the quantitation of amide proton signal intensities. A pair of ^15^N-edited ^1^H 1D spectra was recorded with diffusion periods of either 1.5 s or 2.0 s and bipolar dephasing/rephasing gradients (2 ms duration for each gradient) with strengths ranging from 4.5 to 40.1 G/cm. Very similar answers were obtained for both diffusion times, indicating that convection is not an issue (samples in 3 mm NMR tubes were used). The measurements were performed at 800 MHz, 40 °C.

### Spectral density analysis of CAPRIN1 dynamics in the condensed phase

The spectral density function evaluated at zero frequency, *J*(0) (= 0.4 × *S*^2^*τ*_c_, where *S*^2^ is the amide bond vector order parameter squared and *τ*_c_ is the effective rotational correlation time of the amide in question), was evaluated by measuring relaxation rates for a set of four ^15^N–^1^H operators (^1^H–^15^N longitudinal order, antiphase ^1^H and ^15^N single-quantum coherences, and ^1^H–^15^N multiple quantum coherences with transverse magnetization elements spin-locked in the presence of 15.6 and 2 kHz fields on ^1^H and ^15^N, respectively), along with ^15^N longitudinal relaxation rates (*R*_1_), and ^1^H–^15^N steady-state NOE values. The data were analyzed as described previously (27), assuming a distance between ^1^H and ^15^N spins of 1.02 Å and an axially symmetric ^15^N chemical shift anisotropy tensor with $\left|\sigma_{∥}-\sigma_{⊥}\right|$ = 172 ppm. Relaxation delays used to quantify the decay of the four operators described previously varied from 4 to 40 ms, whereas *R*_1_ rates were measured as described previously (30) using relaxation delays ranging from 10 to 700 ms. ^1^H–^15^N steady-state NOE values were quantified by comparing ^15^N intensities in the presence and absence of ^1^H saturation (30, 31). All pulse sequences were based on TROSY (26). We note that significant millisecond to microsecond timescale exchange contributions to ^15^N *R*_2_ values (>2 s^−1^ in the salt-induced condensed phase and >6 s^−1^ in the ATP-induced condensed phase) were not detected (32). All the measurements were performed at 1 GHz, 40 °C.

### Measuring the concentration of CAPRIN1 in the condensed phase

Condensed phase samples of CAPRIN1 were prepared by mixing ∼7 to 8 mM unlabeled CAPRIN1 with varying volumes of a stock solution of 4 M NaCl, corresponding to bulk concentrations of 400 mM or 1200 mM NaCl, in 25 mM Mes–NaOH buffer, pH 5.5. Protein concentrations in both dilute and condensed phases were obtained by measuring the absorbance at 280 nm after diluting 2 and 5 μl of the condensed and dilute phases into a denaturing buffer (50 mM Tris, 200 mM NaCl, and 3 M guanidine hydrochloride) resulting in sample volumes of 200 and 150 μl, respectively, as described previously (17). The condensed phase solution was carefully sampled using a positive displacement pipette (Eppendorf) (17). We note that the use of the positive displacement pipette was critical for the accurate pipetting of the viscous condensed phase. Concentrations of CAPRIN1 in the condensed and dilute phases are {19.1 ± 1.5 mM} and {3.2 ± 0.6 mM} for the sample prepared with 400 mM bulk NaCl and {38.8 ± 1.6 mM} and {0.25 ± 0.06 mM} for the sample prepared with 1200 mM bulk NaCl. Average values and errors were obtained by multiple measurements on the condensed phase samples (n = 5 and 6 for 400 mM and 1200 mM bulk NaCl, respectively).

## Data availability

The datasets generated during and/or analyzed during the current study are available from the corresponding authors upon reasonable request. Backbone amide chemical shifts of CAPRIN1 have been deposited in the Biological Magnetic Resonance Bank, https://bmrb.io/ (entry ID: 51689).

## Conflict of interest

The authors declare that they have no conflicts of interest with the contents of this article.
